# Development and Validation of a Nomogram to Predict the Risk of Potentially Inappropriate Medication Use in Older Outpatients with Depression

**DOI:** 10.3390/jcm15187084

**Published:** 2026-09-12

**Authors:** Baihui Wu, Zhaoyan Chen, Fangyuan Tian

**Affiliations:** 1 Department of Pharmacy, National Clinical Research Center for Geriatrics, West China Hospital, Sichuan University, Chengdu 610041, China; wubaihui2025@163.com (B.W.); chenzhaoyan1993@163.com (Z.C.); 2 Department of Pharmaceutics, School of Pharmacy, Southwest Medical University, Luzhou 646000, China; 3 Department of Epidemiology and Health Statistics, West China School of Public Health, West China Fourth Hospital, Sichuan University, Chengdu 610041, China

**Keywords:** older adults, depression, potentially inappropriate medication, nomogram, risk prediction model

## Abstract

**Background:** Potentially inappropriate medication (PIM) use among older people is a serious public health problem associated with increased adverse drug events. PIM refers to medications where the adverse risks outweigh the potential benefits. Identifying the risk factors for PIM is essential for optimizing prescription practices and improving patient safety. **Objectives**: To establish a risk prediction model for potentially inappropriate medications (PIMs) in older patients with depression, providing guidance to optimize medication plans, reduce adverse drug reactions, and improve treatment outcomes and quality of life. **Methods**: Prescriptions for depression patients among all hospitals in the Chengdu area were taken as an example. A significant factor influencing PIM risk was identified through univariate and multivariate logistic regression analyses, and a nomogram was constructed. The discrimination and calibration of the model were evaluated via receiver operating characteristic (ROC) curves. **Results**: According to the analysis of the nomogram drawn from the prescriptions of patients with depression, it can be found that the department name, reimbursement, hospital grade, age, number of diseases, sleep disorders, hypertension, cerebrovascular disease and so on each have *p* < 0.05 for PIM. Data from the Chengdu area (*n* = 4629) were divided into a training set (*n* = 3548) and an internal validation set (*n* = 1081), with Zhengzhou data (*n* = 1620) used as the external validation set. ROC curve analysis revealed that the area under the curve (AUC) for the training set was 0.721, that for the internal validation set was 0.668, and that for the external validation set was 0.663. **Conclusions**: The prediction model based on these factors has predictive value for PIM use in older patients with depression, It shows some discriminatory ability, but its external performance is moderate and its calibration is insufficient. Nevertheless, it can be used for preliminary judgment of patients’ medication and as an auxiliary screening tool. Further optimization and prospective validation are still needed.

## 1. Introduction

It is common for older individuals to suffer from multiple diseases and use multiple medications. The interactions between various drugs and the long-term use of medications due to chronic comorbidities make drug interactions complex [1,2,3], which can affect drug efficacy and increase the risk of adverse reactions. This may lead to potentially inappropriate medication (PIM) use. PIM use is defined as a situation where the potential adverse risks of using a medication may outweigh the expected clinical benefits [4].

According to data provided by the World Health Organization (WHO), the global prevalence of late-life depression ranges from 10% to 20%, and in certain regions or specific populations, this proportion may be even greater [5]. In recent years, with the intensification of global population aging, the number of people suffering from late-life depression has been rising steadily. In China, late-life depression is particularly severe, with a depression incidence rate of approximately 10.5% [6]. Although late-life depression is a common mental disorder, there is currently a lack of effective clinical treatment methods, and pharmacotherapy remains the primary intervention. However, many older patients with depression exhibit limited long-term therapeutic effects from drug treatment, poor tolerance to pharmacotherapy, and are prone to adverse reactions. Many of these drugs are PIMs, making older patients with depression a priority population for PIM surveillance.

Although PIM use in older adults has been explored globally, studies specifically targeting older Chinese patients with depression remain scarce, particularly those systematically identifying factors influencing PIM use and developing tailored risk prediction tools. This specific population faces a high risk of PIM use due to multimorbidity, polypharmacy, and age-related physiological changes, making it particularly necessary to investigate the influencing factors of PIM and to develop risk prediction models. Therefore, this study selected the older outpatients with depression as the study population and analyzed PIM usage and influencing factors among older outpatients in China. These findings provide guidance for future efforts to reduce medication risks and improve the medication environment for older patients with depression.

The development of the Chinese PIM standard not only integrated multicenter monitoring data and clinical medication data from China but also involved multiple rounds of expert deliberation and validation in drug classification and risk assessment. It fully considered the disease spectrum characteristics and medication use patterns of older individuals in China, especially the PIM criteria under disease conditions, to ensure the scientific and clinical applicability of the standard [7]. Directly applying foreign PIM standards may lead to inaccurate results. Therefore, this study used the Chinese PIM standard for data screening. The Chinese standard is the dominant standard and is based on the literature or expert consensus. It is usually guided by drugs or diseases and does not require complex clinical judgment when applied. Therefore, there is no strict requirement for the screening personnel, which can be more convenient for allowing patients themselves and their families to judge whether PIM occurs. In this retrospective study, we sought to develop and validate a predictive model of PIM-containing prescriptions for older patients with depression in China by using the Chinese PIM standard.

## 2. Materials and Methods

### 2.1. Data Sources

The data for this study were derived from the “Hospital Prescription Analysis Collaboration Project” of the Chinese Pharmaceutical Association. Initially, a cluster sampling method was employed to select the prescription medication data of older patients with depression from January to December 2021 in Chengdu and Zhengzhou. Prescription medication data refers to all medications prescribed during a single outpatient visit (regardless of whether they were dispensed or picked up). These are hospitals with comprehensive outpatient departments and electronic information systems in Chengdu and Zhengzhou. The data extracted included patient personal information (age and sex), medication information (drug name and cost), and diagnostic information. Specifically, this study focused on the prescription data of older patients with depression from the Chengdu and Zhengzhou regions from January to December 2021.

### 2.2. Inclusion and Exclusion Criteria

The inclusion criteria were as follows: (1) aged ≥ 65 years. (2) Outpatient data. (3) A definite diagnosis of depression was formally made by an attending psychiatrist or a professional neurologist in a routine outpatient setting, which was based on the Diagnostic and Statistical Manual of Mental Disorders, Fifth Edition (DSM-5) and the International Classification of Diseases (10th Edition) (ICD-10).

The exclusion criteria were as follows: (1) Only “depressive state” or “emotional disorder” was recorded as the diagnosis without a specific ICD-10 diagnosis or with incomplete information records. (2) No pharmacotherapy was used. This study was conducted according to the guidelines of the Declaration of Helsinki and approved by the Sichuan University West China Hospital Research Ethics Board (protocol code 2024/810, date of approval: 30 April 2024).

### 2.3. Data Cleaning

Data cleaning involved the removal of records where the patient’s age was less than 65 years, or where prescription information was incomplete or missing, or where the prescribed medication was a topical preparation or solvent. Additionally, prescription records with the same case number and date were merged and counted as a single case.

### 2.4. Prescription Identification and Statistics

After data cleaning, prescriptions were identified, and their quantities were counted via “prescription codes”. Each prescription code corresponds to a single prescription record and is unique, covering all medication records from a patient’s single visit.

When determining and calculating the frequency of PIM-containing prescriptions, the following principles were applied: (1) if a prescription contained at least one medication meeting any criterion of the 2017 Chinese PIM standard, including both drug-specific and disease-state-specific criteria, it was classified as a PIM-containing prescription and counted as one PIM occurrence to calculate the PIM detection rate, which served as the primary outcome for all statistical models; (2) if a prescribed medication met multiple criteria of the PIM evaluation standard, the occurrences were cumulatively counted to calculate the total number of PIMs detected per prescription, which was reported for descriptive purposes only and not entered into any statistical model.

### 2.5. Evaluation Criteria

This was a retrospective, multicentre, cross-sectional analysis of outpatient prescription records obtained from the Hospital Prescription Analysis Collaboration Project of the Chinese Pharmaceutical Association.

The Chinese PIM standard consists of two parts. The first part includes 13 major categories of 72 drugs/classes of drugs, such as drugs for the nervous system, cardiovascular system, and antiallergic system. Each drug/class of drugs is accompanied by 1 to 6 risk points for drug use. These risk points focus on severe, frequent, and common risks. Some drugs also include additional points of concern and medication suggestions, which help clinicians and pharmacists accurately and quickly identify the risks of each drug, making them more practical and referable. On the basis of the average score of expert-evaluated indicators, drugs are classified by risk intensity: (1) High-risk medications should be avoided by older individuals. (2) Low-risk medications should be used with caution by older patients.

Part 2 includes 44 types/classes of drugs across 27 disease states, requiring joint evaluation of both the disease and the drug to determine whether the medication is a PIM. On the basis of medication frequency (total annual consumption of a drug divided by its defined daily dose), the 44 types/classes of drugs are categorized into Class A and Class B alert medications: (1) Class A warning drugs have a usage frequency ≥ 3000, such as nonsteroidal anti-inflammatory drugs used for heart failure. (2) Class B warning drugs have a usage frequency < 3000, such as triazolam used for insomnia. Organizing medications on the basis of disease state facilitates the determination of whether a PIM is being used, making this standard more clinically instructive.

### 2.6. Statistical Analysis

In this study, the construction of a PIM risk prediction model for older patients with depression was a typical binary classification task. Therefore, this study employed logistic regression to construct a risk prediction model. Candidate variables are shown in Table 1. All variables were transformed into categorical variables for analysis. For continuous variables, such as age, the coding was discretized according to the previous literature or medical reference value ranges; patients aged 65–79 years were coded as 0, and those aged ≥80 years as 1. For unordered categorical variables, such as the reimbursement type, a binary or multi-category approach was used.

In order to select statistically significant predictors, binary logistic regression was used for univariate analysis to screen for factors with *p* < 0.05, and then all variables with *p* < 0.05 in univariate analysis were included in multivariate logistic regression analysis without other variable selection procedures. The results were presented as odds ratios (ORs) and 95% confidence intervals (CIs). The OR measures the strength of the association between a certain influencing factor and the outcome event. *p* < 0.05 was considered statistically significant. The greater the OR, the higher the risk of PIM. Variables with statistical significance (*p* < 0.05) in the multivariate logistic regression analysis were used to construct a nomogram via R Studio version 4.2.0. The model training set comprised outpatient prescription data from the second to fourth quarters of 2021 in Chengdu. The first quarter includes the Spring Festival holiday, during which outpatient volumes fluctuate and patients’ healthcare-seeking behavior may change. It also encompassed drug policy adjustments and medical insurance settlement cycles at the beginning of the year, which may influence physician prescribing choices, as well as seasonal respiratory infections prevalent in winter and spring that may affect concomitant medication patterns. Consequently, this period was not used for model fitting. Despite these specificities, this cohort still represented an independent temporal window that can verify the model’s stability over time, and was therefore used as a temporally distinct validation cohort. Data from the 2nd to 4th quarters cover spring, summer, and autumn, and can more comprehensively reflect the disease spectrum and medication characteristics under different climatic conditions. This makes it easier to identify the influencing factors of PIM-containing prescriptions in older patients with depression. For external validation, data from the corresponding period in Zhengzhou were selected. The city is located in the central region. The medical system and prescription culture were different from Chengdu, and its sample size was similar to that of Chengdu, which could effectively test the external applicability of the model and enhance the robustness of the fitted model.

The goodness of fit of the model was evaluated by receiver operating characteristic (ROC) curve and area under the curve (AUC). An AUC > 0.7 is generally considered to indicate good discriminatory power [8]. The Hosmer–Lemeshow test is used to assess the calibration ability of the model, that is, the consistency between the predicted probabilities of the model and the actual observed results. *p* > 0.05 in the test is generally considered to indicate good calibration of the model [9].

## 3. Results

### 3.1. Descriptive Statistics

**Figure 1:**
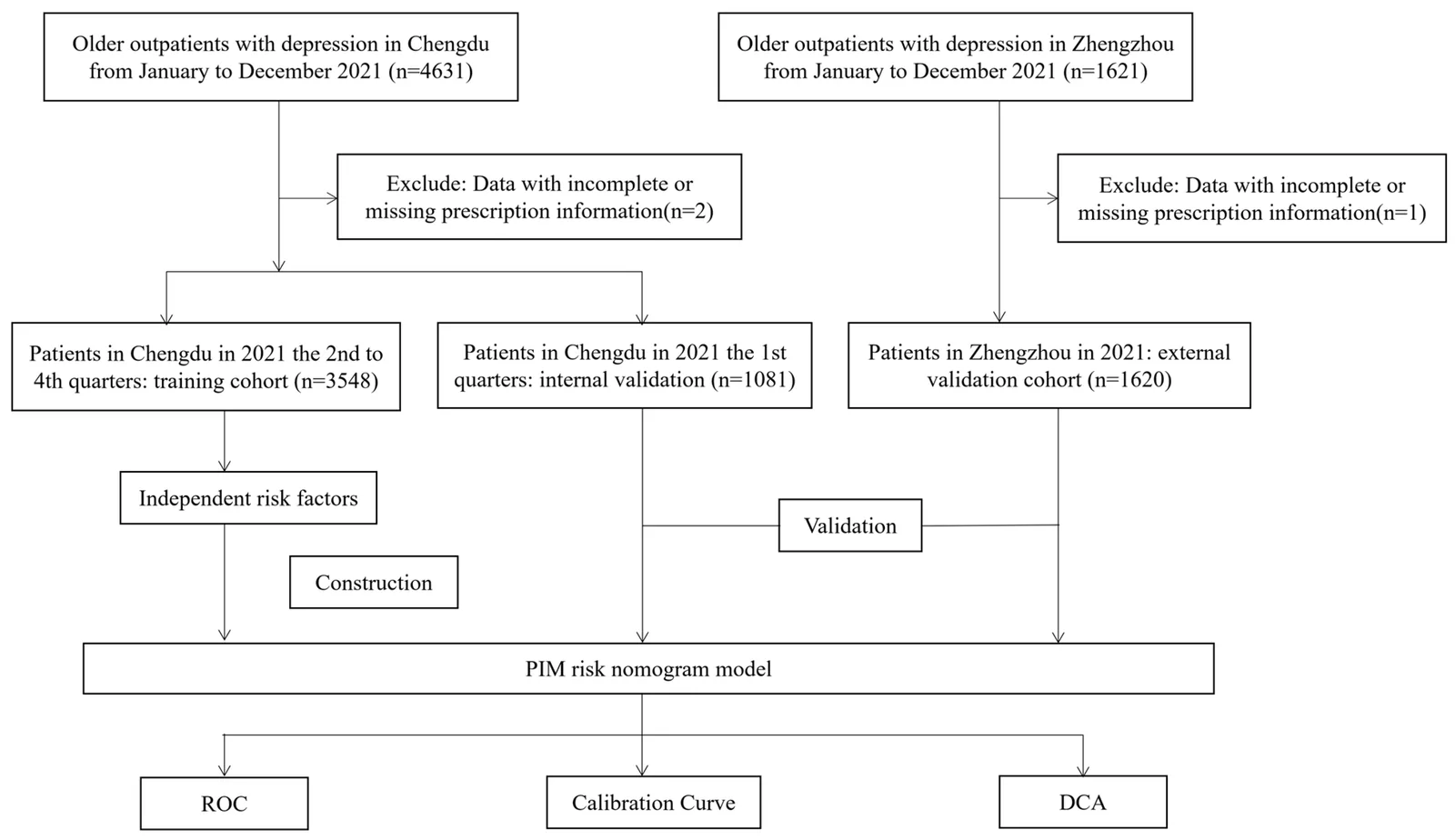
Flow diagram of older depressed patients’ prescriptions identified in the study. ROC, receiver operating characteristic; DCA, decision curve analysis.

This study included a total of 6249 prescriptions for older patients with depression. In the training set (*n* = 3548), there were 1615 prescriptions (45.5%) for older patients with depression involving PIM. In the internal validation set (*n* = 1081), 497 prescriptions (46.0%) involving PIMs were included. In the external validation set (*n* = 1620), there were 1015 prescriptions (62.7%) involving PIMs. The prescription selection process is depicted in Figure 1.

Prescriptions for older patients with depression aged ≥80 years accounted for 24.2% (*n* = 1512) of the sample. Among the prescriptions for older patients with depression, 78.16% (*n* = 4884) had multiple chronic conditions, and 16.96% (*n* = 1060) included ≥5 medications. Additionally, anxiety symptoms were present in 57.67% (*n* = 3604) of the prescriptions, and insomnia symptoms were present in 28.9% (*n* = 1806) of the prescriptions. The basic characteristics of the patients are shown in Table 1. The basic characteristics of patients’ prescriptions were included in the range of candidate factors.

### 3.2. Influencing Factors

The results of the univariate analysis for older patients’ prescriptions for depression revealed that the number of comorbid diseases, age, the number of medications, type of reimbursement, department name, hospital level, anxiety disorder, sleep disorder, diabetes, hypertension, osteoporosis, and cerebrovascular disease were all significantly associated with the occurrence of PIM usage in older patients with depression (*p* < 0.05).

The variables with *p* < 0.05 from the univariate analysis were subsequently included in the multivariate logistic regression analysis. The results indicated that the number of comorbid diseases, type of reimbursement, department name, hospital level, anxiety disorder, sleep disorder, diabetes, hypertension, age, and cerebrovascular disease were significant factors influencing the use of PIM-containing prescriptions in older patients with depression (*p* < 0.05). The detailed results are shown in Table 2.

### 3.3. A Nomogram for Predicting PIM

**Figure 2:**
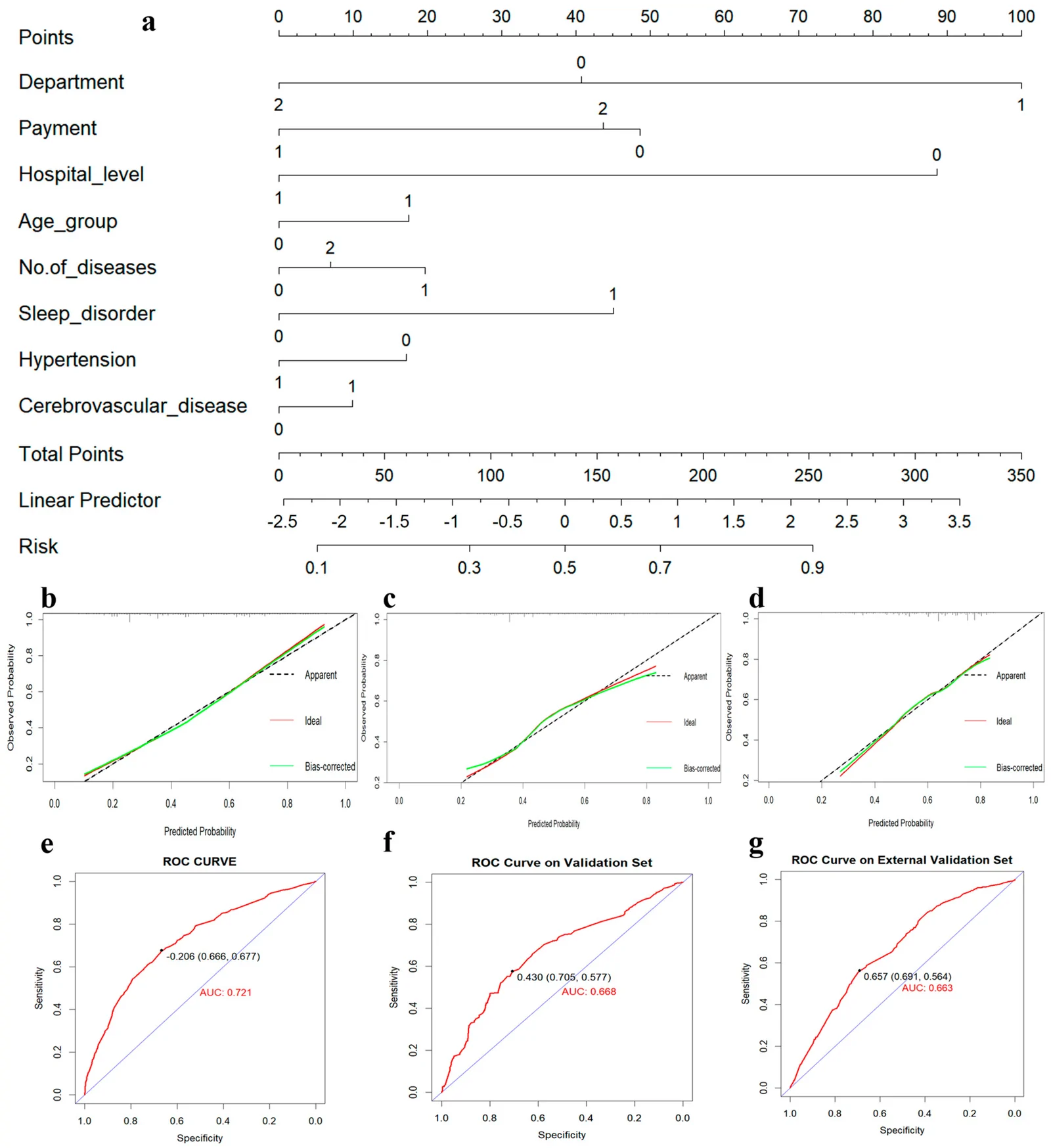
Nomogram for PIM in older patients’ prescriptions for depression in Chengdu (**a**); calibration curve for the training (**b**) and validation (**c**,**d**) cohorts for PIM; ROC curve for the training (**e**) and validation (**f**,**g**) cohorts for PIM.

Using multivariate logistic regression, the variables were screened by *p* value, OR value and clinical practical factors. According to the above univariate analysis regression results, only variables with *p*-values > 0.05 were retained. The following variables were selected to construct the prediction model: department name, payment, hospital level, age, number of diseases, sleep disorders, hypertension, and cerebrovascular disease. A nomogram was then developed on the basis of these variables, and the results are shown in Figure 2a.

The AUC value of the training set for the PIM model in older patients’ prescriptions for depression was 0.72 (0.698–0.744); an AUC > 0.7 is generally considered to indicate good discrimination [10]. The Hosmer–Lemeshow test value was 0.29. Usually, a Hosmer–Lemeshow > 0.05 is considered to indicate good calibration [9], thus showing that the training set model had good discrimination and calibration. The results are shown in Figure 2b,e.

The model was applied to the internal validation set for testing. The internal validation results revealed that the AUC value of the logistic regression model was 0.668 (0.641–0.695), indicating acceptable discriminatory ability. The Hosmer–Lemeshow test value was less than 0.05, suggesting that the model calibration was not ideal. The calibration curve is shown in Figure 2c, and the ROC curve is shown in Figure 2f.

The model was applied to the external validation set to evaluate its generalizability and extrapolation capability. The external validation results revealed that the AUC value of the logistic regression model was 0.663 (0.636–0.690), indicating moderate discriminatory ability. The Hosmer–Lemeshow test value was less than 0.05, suggesting that the model calibration was not ideal. The calibration curve is shown in Figure 2d, and the ROC curve is shown in Figure 2e.

**Figure 3:**
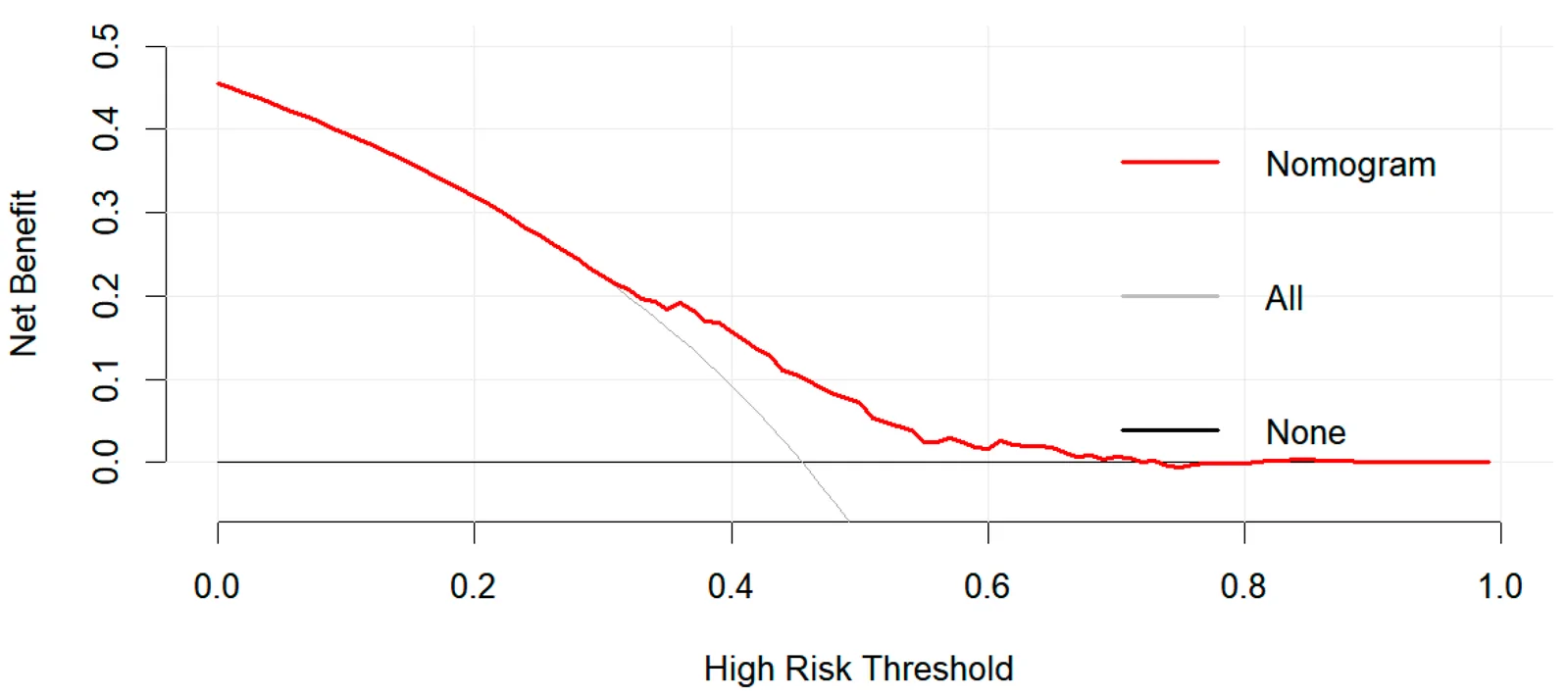
Decision curve analysis for PIM in older patients’ prescriptions for depression.

For PIM in older patients’ prescriptions for depression, DCA revealed that the nomogram has a greater net benefit than strategies based on considering either all patients or no patients for intervention at risk thresholds up to approximately 70% (Figure 3).

## 4. Discussion

To the best of our knowledge, this study represents the first attempt to assess the factors influencing potentially inappropriate medication (PIM) use among older outpatients in China, filling the research gap of insufficient targeted studies on older Chinese patients with depression. Based on the Chinese PIM criteria, we identified predictors for a PIM risk prediction model in this population, thus providing a targeted tool for clinical PIM screening in this specific group. Regarding the performance of the constructed nomogram, its moderate discriminative ability in the training set indicates that the model can capture some key prescribing patterns related to PIM-containing prescriptions. However, its suboptimal performance in both internal and external validation sets, especially the poor calibration, reflects obvious limitations in the model’s generalizability and reliability. Therefore, the model can be used as an auxiliary tool for screening but is not recommended as a standalone basis for clinical decision-making.

This study selected the prescription data of the first quarter for internal verification. Although the first quarter may be different from other quarters because of the Spring Festival, we believe that this is a stress test for the universality of the model. Models that perform well on datasets with significant seasonal differences are more likely to be extended to other clinical environments with different prescription cultures. In our study, the performance of the model in Q1 was only slightly lower than that in the training set, indicating that the model did not seriously overfit the Q2-Q4 training data. Nevertheless, given the suboptimal calibration in the external validation set, this generalizability remains to be further verified in more diverse clinical settings. Our findings confirmed that age, number of comorbidities, and polypharmacy were associated with PIM-containing prescriptions, which is consistent with the findings of Tian et al. [11,12]. Advanced age and multiple chronic diseases increase the likelihood of receiving multiple drug regimens, thereby elevating the risk of inappropriate prescribing. According to a study by Tian et al. [13,14], PIM-containing prescriptions were associated with sex, polypharmacy, and multiple chronic diseases. Research by other scholars [15] has revealed that polypharmacy is positively correlated with the proportion of PIM prescriptions detected, which is consistent with the results of this study. Owing to the physiological decline caused by aging and the presence of chronic diseases [16], older patients are highly susceptible to polypharmacy.

Nevertheless, the present study found that although the number of comorbidities was a variable that helped predict PIM-containing prescriptions, the influence was most pronounced among patients with two to four comorbidities. The non-significant result observed in the highest comorbidity subgroup may seem counterintuitive. One plausible explanation is that patients with five or more chronic conditions generally receive highly complex medication regimens and are under closer monitoring by geriatricians or clinical pharmacists. Such intensive surveillance may conversely lower the prevalence of PIMs, as prescribers exercise greater caution and conduct more frequent medication reviews. In addition, patients with an extremely high comorbidity burden may have shorter life expectancies or higher hospitalization rates, resulting in fewer eligible outpatient prescriptions and a lower observed PIM rate in the outpatient setting [17]. Although there is evidence of an increased risk of PIM-containing prescriptions in older women [13,18,19], some studies support the current analysis that there is no significant association between female gender and PIM exposure [18]. A potential reason is that our study population was restricted entirely to patients with depression. Depression is known to be more prevalent and severe in females [20], and many antidepressants classified as PIMs (e.g., amitriptyline, paroxetine) are commonly prescribed for both sexes [21]. This high baseline exposure to potentially inappropriate antidepressants may attenuate gender-related differences in PIM risk, thereby diminishing the independent effect of sex on PIM-containing prescriptions.

PIMs are also related to the patient’s disease status and department. Alhawassi et al. [22] showed that polypharmacy and the presence of certain chronic comorbidities are associated with a greater risk of PIM-containing prescriptions in older patients. Similar conclusions have been drawn by other scholars [23,24,25]. Therefore, age and the number of diseases affect not only the types and quantities of medications used but also the factors influencing PIM-containing prescriptions in older patients. Clinically, it is important to focus on older patients with multiple diseases, especially those who are older, and to strengthen the assessment and monitoring of medication use. Different PIM evaluation criteria are formulated on the basis of national conditions and medication use in various countries, and there are differences in content, number of items, and target populations. A comparison of the 2019 Beers criteria and the 2014 STOPP criteria with the Chinese PIM standard revealed that the 2019 Beers and 2014 STOPP criteria apply to patients aged ≥ 65 years, whereas the Chinese PIM standard applies to patients aged ≥ 60 years. The 2019 Beers criteria list a total of 99 PIMs, the 2014 STOPP criteria contain 81 PIMs, and the Chinese PIM standard includes 106 PIMs. The 2019 Beers criteria cover five major categories: medications that older adults should avoid, medications to be used with caution in certain disease states, medications to be avoided in renal impairment, drug–drug interactions, and drug–disease interactions. The 2014 STOPP criteria are classified by physiological systems and involve 13 categories, including the respiratory and central nervous systems, focusing mainly on PIMs in specific disease states, whereas the Chinese PIM standards are divided into two categories: medications to be avoided and PIMs in disease states. Therefore, the Chinese PIM fully considers the disease spectrum and prescribing habits of older individuals in China. It includes criteria for judging PIMs under disease conditions and has undergone multiple rounds of expert deliberation and validation in drug classification and risk assessment, ensuring the scientific and practical nature of the PIM items. Owing to cultural differences and medical backgrounds, standards developed for other populations may not be suitable for the older population in China. Moreover, standards developed for other populations may not be comprehensive in their updates and revisions. Therefore, this study used the Chinese PIM standard for analysis. Moreover, the Chinese PIM standard is an explicit standard that is based on the literature and expert consensus. It is usually guided by drugs or diseases and offers high accuracy with no need for complex clinical judgment in application.

Logistic regression models are now widely applied in various medical fields, including disease diagnosis, medical decision-making, and predicting the risk of adverse outcomes after medication use. Previous researchers have also constructed logistic regression models to predict PIMs. Tian et al. [26] used logistic regression to construct a nomogram to predict the risk of PIM-containing prescriptions in older patients with lung cancer, and the nomogram was constructed using six influencing factors: payment, charge, disease, polypharmacy, insomnia, and pain. The results of the ROC curve analysis were satisfactory, and both the internal and external validation results were good. The nomogram demonstrated a high net benefit in DCA. This finding indicates that the use of a nomogram for analyzing influencing factors is valuable. When similar methods are used to study patients with other diseases, attention should be given to the selection of influencing factors, and a reasonable judgment should be made on whether the use of multiple medications by older patients with multimorbidity increases the risk of PIM. These studies focused on patients with lung cancer, and there were no reports on the construction of PIM risk prediction models for older patients with depression. Moreover, most of the above studies were single-center studies, while this study is based on multicenter data and uses the Chinese PIM standard, which is more suitable for domestic use. Therefore, we conclude that a PIM risk prediction model tailored to specific populations is essential.

Using this nomogram, clinicians can facilitate the targeted screening of older adults with depression who are vulnerable to PIM. For example, this tool can be used to strengthen PIM screening training for older patients with depression. For those with comorbid sleep disorders and hypertension, it is recommended to optimize the medication regimen to avoid PIM use. Overall, this tool can help doctors to screen for early inappropriate medication use, so that high-risk individuals can be identified early. By providing clinicians with targeted clinical intervention recommendations, it serves as a valuable reference for clinical practice.

This study has several limitations that warrant attention. First, only the Chinese PIM criteria were applied; no comparison was made with the STOPP or 2019 Beers criteria, so global benchmarks for PIM are unavailable. Second, although hospital identifiers were available in the database, the absence of unique patient identifiers precluded the identification of repeated prescriptions from the same patient and the application of statistical methods accounting for patient-level clustering. Consequently, the independence of observations could not be confirmed, which may have resulted in underestimated standard errors, biased parameter estimates, and consequently diminished reliability of statistical inferences and study conclusions. In addition, we could not exclude the possibility that the same patient contributed prescriptions to both the training and validation sets, and the validation results should be interpreted with caution. Owing to these data constraints, we were unable to employ mixed-effects models to account for repeated measurements within individuals. Regarding hospital-level clustering, this study did not adopt cluster-robust standard errors or multilevel models to account for the clustering of prescriptions within hospitals in the primary analysis, which is also a limitation. Third, the data pertained to older outpatients with depression and were derived from a retrospective database analysis. Although previous studies have established that medication adherence is central to chronic disease management [27], the limited outpatient follow-up precluded assessment of patients’ medication adherence, its association with payment method, and the presence of drug–drug interactions. Consequently, the real-world clinical impact remains uncertain.

This model was trained on the Chengdu cohort with a low PIM detection rate (46%), whereas the Zhengzhou validation cohort exhibited a higher detection rate (62.7%); although the PIM evaluation criteria and patient inclusion/exclusion criteria were completely consistent between the two cities, subtle differences in local clinical prescribing habits may have led to discrepant PIM detection rates, resulting in misclassification. Furthermore, the predicted probabilities systematically underestimated the actual PIM risk in Zhengzhou, yielding a suboptimal trade-off between sensitivity and specificity and ultimately compromising external validation performance. The samples were derived only from Chengdu and Zhengzhou, imposing geographical constraints. Future studies should expand the sampling area and conduct prospective research; moreover, rigorous validation approaches such as random splitting or k-fold cross-validation are recommended to comprehensively evaluate the influence of seasonal variation and temporal bias on model generalizability.

There are many potential reasons for the decrease in AUC and poor calibration of the model in external verification. It may be due to the imbalance of samples, which biases the model towards the majority class during the training process, resulting in the inconsistency between predicted probabilities and observation frequency. It may also be due to the fact that only the significance is considered in data screening, and the predictors may improve the prediction even if the individual correlation is not significant, while the selection based on *p*-value may lead to unstable coefficients and optimistic performance estimation. An improper modeling approach may not fully capture the complex nonlinear relationships between influencing factors and PIM-containing prescriptions. Differences in prescribing habits, patient disease spectrum, heterogeneity in healthcare quality, and differences in PIM detection rates between the two cities may lead to poor external validation performance.

Given the model’s limitations, it should only be used as a low-threshold screening tool in settings similar to the derivation cohort, and further optimization is imperative before clinical implementation. Future studies should focus on improving model performance by addressing sample imbalance using techniques such as oversampling or undersampling, comparing multiple machine learning approaches, for example, LASSO regularization and random forest, and extending the time span of validation data to avoid temporal bias. These optimizations will enhance the model’s discriminative ability, calibration, and generalizability, thereby facilitating its clinical application. Additionally, developing a dynamic web-based nomogram for real-time individualized calculation would help clinicians identify decision intervals with high uncertainty, enabling multi-scenario adaptation.

## 5. Conclusions

On the basis of real-world prescription data, this study used logistic regression to construct a PIM risk prediction model for older patients with depression according to the Chinese PIM standard. This model serves as an auxiliary screening tool with moderate discriminatory ability; although it cannot be directly used for clinical decision-making, it may provide a preliminary reference for doctors and clinical pharmacists and offers reliable evidence for the medication safety of older patients with depression. The results show that although the external calibration was suboptimal, local recalibration is warranted before the model can assist medical staff in different regions and different levels of medical institutions in medication review for older patients with depression, and assist clinicians in drug analysis. This nomogram enables clinicians to preliminarily identify older adults with depression at high risk for PIM, and provides a foundation for further optimizing and developing targeted predictive models with improved discrimination and calibration in future research. This research has clinical significance for improving rational medication use guidelines for older patients with depression in China.

## Figures and Tables

**Table 1:** Basic characteristics of the training, internal validation, and external validation methods.

Characteristics	Training (%)	Internal Validation (%)	External Validation (%)
PIM			
Yes	1615 (45.5%)	497 (46.0%)	1015 (62.7%)
No	1933 (54.5%)	584 (54.0%)	605 (37.3%)
Hospital level			
2nd	68 (1.9%)	31 (2.9%)	73 (4.5%)
3nd	3480 (98.1%)	1050 (97.1%)	1547 (95.5%)
Department			
Other	2657 (74.9%)	808 (74.7%)	1241 (76.6%)
Psychiatry	743 (20.9%)	233 (21.6%)	228 (14.1%)
Geriatrics	148 (4.2%)	40 (3.7%)	151 (9.3%)
Sex			
Female	2379 (67.1%)	705 (65.2%)	991 (61.2%)
Male	1169 (32.9%)	376 (34.8%)	629 (38.8%)
Age group			
65–79	2890 (81.5%)	853 (78.9%)	994 (61.4%)
≥80	658 (18.5%)	228 (21.1%)	626 (38.6%)
No. of diseases			
1	783 (22.1%)	234 (21.6%)	348 (21.5%)
2–4	1758 (49.5%)	571 (52.8%)	833 (51.4%)
≥5	1007 (28.4%)	276 (25.5%)	439 (27.1%)
No. of medications			
1–4	2969 (83.7%)	924 (85.5%)	1296 (80.0%)
≥5	579 (16.3%)	157 (14.5%)	324 (20.0%)
Payment			
Free	1218 (34.3%)	417 (38.6%)	169 (10.4%)
Partial Fee	1368 (38.6%)	352 (32.6%)	447 (27.6%)
Full fee	962 (27.1%)	312 (28.9%)	1004 (62.0%)
Disease			
Anxiety			
Yes	1971 (55.6%)	588 (54.4%)	1045 (64.5%)
No	1577 (44.4%)	493 (45.6%)	575 (35.5%)
Sleep disorder			
Yes	1012 (28.5%)	315 (29.1%)	479 (29.6%)
No	2536 (71.5%)	766 (70.9%)	1141 (70.4%)
Dementia			
Yes	78 (2.2%)	15 (1.4%)	85 (5.2%)
No	3470 (97.8%)	1066 (98.6%)	1535 (94.8%)
Hypertension			
Yes	836 (23.6%)	251 (23.2%)	383 (23.6%)
No	2712 (76.4%)	830 (76.8%)	1237 (76.4%)
Coronary heart disease			
Yes	324 (9.1%)	101 (9.3%)	325 (20.1%)
No	3224 (90.9%)	980 (90.7%)	1295 (79.9%)
Diabetes			
Yes	389 (11.0%)	104 (9.6%)	202 (12.5%)
No	3159 (89.0%)	977 (90.4%)	1418 (87.5%)
Arthritis			
Yes	54 (1.5%)	13 (1.2%)	35 (2.2%)
No	3494 (98.5%)	1068 (98.8%)	1585 (97.8%)
Hyperlipidemia			
Yes	188 (5.3%)	57 (5.3%)	96 (5.9%)
No	3360 (94.7%)	1024 (94.7%)	1524 (94.1%)
Osteoporosis			
Yes	119 (3.4%)	20 (1.9%)	38 (2.3%)
No	3429 (96.6%)	1061 (98.1%)	1582 (97.7%)
Cerebrovascular disease			
Yes	542 (15.3%)	154 (14.2%)	136 (8.4%)
No	3006 (84.7%)	927 (85.8%)	1484 (91.6%)

**Table 2:** Results of univariate and multivariate logistic regression analyses.

Univariate Analysis		*p*	Multivariate Analysis		*p*
Characteristics	OR (95% CI)		Characteristics	OR (95% CI)	
Hospital level			Hospital level		
2nd	Reference		2nd	Reference	
3nd	0.251 (0.138–0.431)	<0.001	3nd	0.193 (0.104–0.341)	<0.001
Department			Department		
Other	Reference		Other	Reference	
Psychiatry	2.563 (2.167–3.038)	<0.001	Psychiatry	2.812 (2.325–3.406)	<0.001
Geriatrics	0.357 (0.233–0.530)	<0.001	Geriatrics	0.483 (0.310–0.732)	<0.001
Sex			Sex	-	
Female	Reference		Female		
Male	0.949 (0.825–1.093)	0.468	Male		
Age group			Age group		
65–79	Reference		65–79	Reference	
≥80	1.201 (1.014–1.423)	0.038	≥80	1.414 (1.168–1.714)	<0.001
No. of diseases			No. of diseases		
1	Reference		1	Reference	
2–4	1.316 (1.110–1.560)	0.002	2–4	1.444 (1.165–1.792)	<0.001
≥5	1.020 (0.844–1.233)	0.837	≥5	1.273 (0.979–1.658)	0.072
No. of medications			No. of medications		
1–4	Reference		1–4	Reference	
≥5	0.835 (0.697–0.999)	0.049	≥5	0.814 (0.650–1.017)	0.071
Payment			Payment		
Free	Reference		Free	Reference	
Partial Fee	0.306 (0.260–0.360)	<0.001	Partial Fee	0.386 (0.322–0.461)	<0.001
Full fee	0.843 (0.711–0.999)	0.049	Full fee	0.874 (0.710–1.075)	0.201
Disease			Disease		
Anxiety			Anxiety		
No	Reference		No	Reference	
Yes	0.839 (0.734–0.958)	0.01	Yes	0.784 (0.674–0.913)	0.002
Sleep disorder			Sleep disorder		
No	Reference		No	Reference	
Yes	2.421 (2.086–2.813)	<0.001	Yes	2.422 (2.032–2.890)	<0.001
Dementia			Dementia	-	
No	Reference		No		
Yes	0.974 (0.617–1.526)	0.908	Yes		
Hypertension			Hypertension		
No	Reference		No	Reference	
Yes	0.813 (0.695–0.951)	0.01	Yes	0.816 (0.669–0.994)	0.044
Coronary heart disease			Coronary heart disease	-	
No	Reference		No		
Yes	0.796 (0.630–1.003)	0.054	Yes		
Diabetes			Diabetes		
No	Reference		No	Reference	
Yes	0.497 (0.395–0.621)	<0.001	Yes	0.495 (0.379–0.645)	<0.001
Arthritis			Arthritis	-	
No	Reference		Yes		
Yes	0.700 (0.395–1.210)	0.209	No		
Hyperlipidemia			Hyperlipidemia	-	
No	Reference		No		
Yes	0.766 (0.566–1.032)	0.082	Yes		
Osteoporosis			Osteoporosis		
No	Reference		No	Reference	
Yes	0.488 (0.323–0.721)	<0.001	Yes	0.708 (0.451–1.091)	0.124
Cerebrovascular disease			Cerebrovascular disease		
No	Reference		No	Reference	
Yes	1.205 (1.003–1.447)	0.046	Yes	1.267 (1.024–1.567)	0.029

## Data Availability

All data generated or analyzed during this study can be obtained from the corresponding author upon inquiry due to privacy and ethical restrictions.
